# Supraventricular Tachycardia Induced by Mediastinal Irritation From a Chest Tube: A Rare and Overlooked Complication

**DOI:** 10.7759/cureus.84145

**Published:** 2025-05-15

**Authors:** Kasun Maduranga, Hiruni Nimesha, Arthihai Srirangan, Eshantha Perera

**Affiliations:** 1 Department of Pulmonary Medicine, National Hospital for Respiratory Diseases, Welisara, LKA

**Keywords:** emphysema subcutaneous, intercostal (ic) tube, mechanical irritation, recurrent pneumothorax, supraventricular tachycardia (svt)

## Abstract

Supraventricular tachycardia (SVT) is commonly associated with intrinsic cardiac or metabolic causes. However, mechanical irritation of mediastinal structures from thoracic intervention is an uncommon and often overlooked trigger. Irritation of the pericardium or nearby autonomic fibers can disrupt normal conduction pathways and provoke reentrant arrhythmias such as SVT.

We report a case of a 40-year-old male with diabetes mellitus who initially underwent right-sided intercostal (IC) tube insertion for a pneumothorax. Following accidental tube dislodgement, he developed recurrent pneumothorax and extensive subcutaneous emphysema, requiring multiple additional IC tubes. During the lung re-expansion phase, the patient developed SVT. Imaging revealed that a left-sided chest tube was near the mediastinum, suggesting mechanical irritation as the likely precipitating factor. The arrhythmia was managed successfully with intravenous adenosine, followed by a short course of oral verapamil for rhythm control.

This case highlights a rare but significant complication of chest tube insertion. Mechanical irritation of the mediastinum should be considered a potential cause of new-onset arrhythmia following thoracic interventions. Early recognition and proper tube positioning are essential to prevent adverse outcomes.

## Introduction

Supraventricular tachycardia (SVT) is a rapid cardiac arrhythmia originating above the ventricles, often triggered by structural heart disease, electrolyte imbalances, or autonomic influences [1]. While SVT is commonly encountered in clinical practice, mechanical irritation of the mediastinal structures as a cause remains a rare and under-recognized phenomenon. Thoracic intervention, including intercostal tube insertion, can occasionally provoke arrhythmic events through mechanical stimulation of the pericardium or nearby autonomic fibers [2].

Here, we explore an unusual instance of SVT that occurred following thoracic intervention in a patient with recurrent pneumothorax and subcutaneous emphysema. While SVT is common, cases triggered by mechanical irritation from intercostal tubes are infrequently reported in the literature, making this presentation clinically relevant. Our patient’s underlying diabetes mellitus may have contributed to autonomic instability, potentially lowering the threshold for arrhythmia. The close temporal relationship between left-side chest tube insertion and SVT onset, without prior cardiac history or metabolic derangement, reinforces the suspicion of a causal link between mediastinal irritation and arrhythmogenesis. This case underscores the need for clinicians to maintain a high index of suspicion for mechanical triggers of arrhythmia, especially in the setting of repeated pleural interventions.

## Case presentation

A 40-year-old Sri Lankan patient developed a fever, cough, and difficulty breathing over one month. He was initially admitted to a local hospital, where a chest X-ray revealed a right-sided pneumothorax. His past medical history included diabetes mellitus for four years. He was subsequently transferred to the National Hospital for Respiratory Diseases, Welisara, Sri Lanka.

On admission, he had a well-functioning right-sided intercostal tube. He was not in respiratory distress; his respiratory rate was 18 breaths per minute, oxygen saturation was 97% on room air, pulse rate was 89 beats per minute, and his blood pressure was 110/70 mmHg.

**Figure 1:**
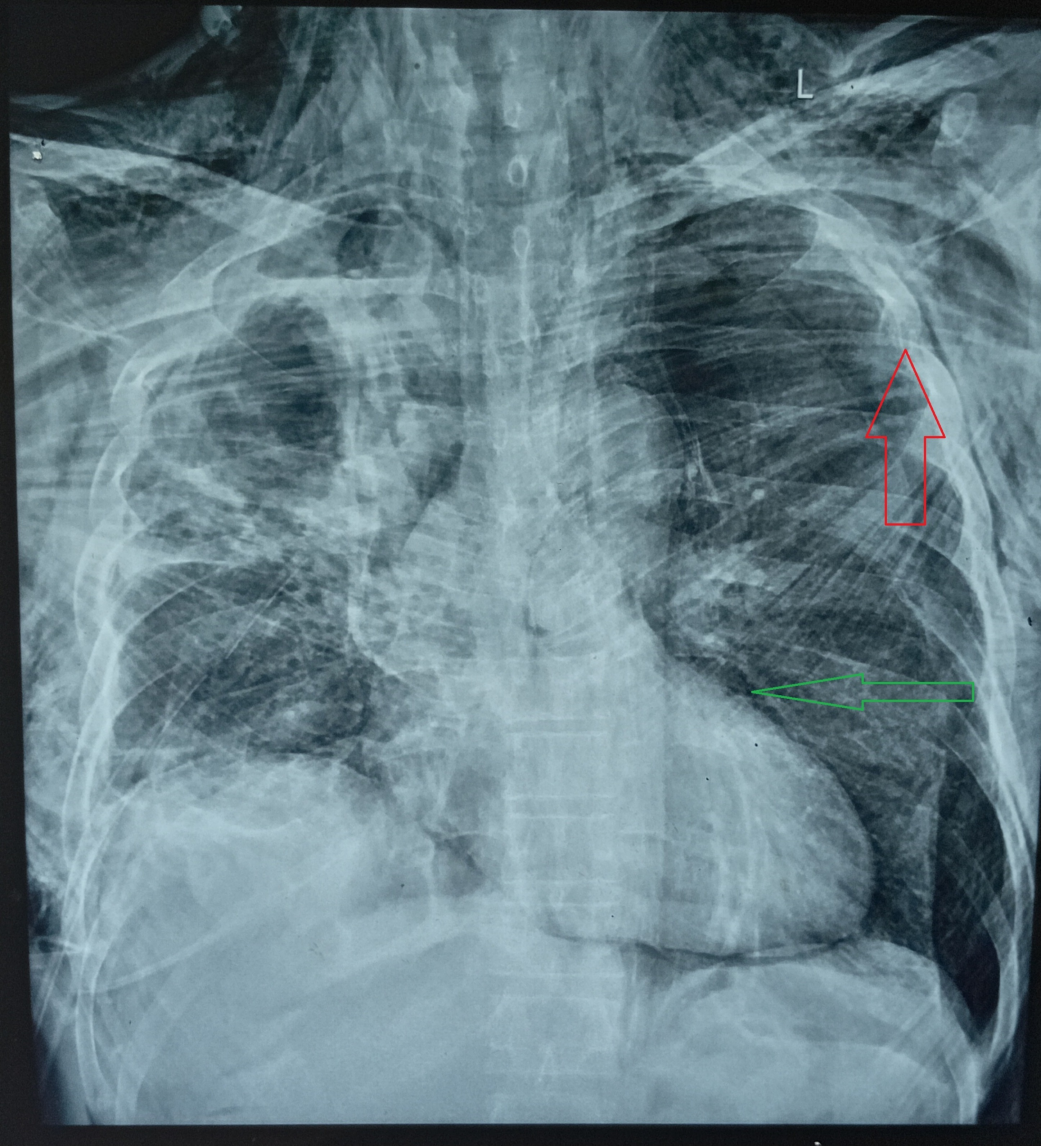
Ginkgo leaf sign (red arrow) suggestive of extensive subcutaneous emphysema and pneumomediastinum (green arrow).

On the second day of his hospital stay, the patient accidentally removed his right-sided intercostal tube. He subsequently developed worsening dyspnea. On examination, he was noted to have palpable subcutaneous crepitus over his upper body and mild soft tissue swelling. A chest X-ray demonstrated the “Ginkgo leaf sign” indicative of extensive subcutaneous emphysema and pneumomediastinum (Figure 1). Three intercostal tubes were inserted, two on the right side for decompression and one on the left side due to radiological evidence of subcutaneous air extending across the mediastinum and concern for early contralateral involvement and mediastinal shift. 

**Figure 2:**
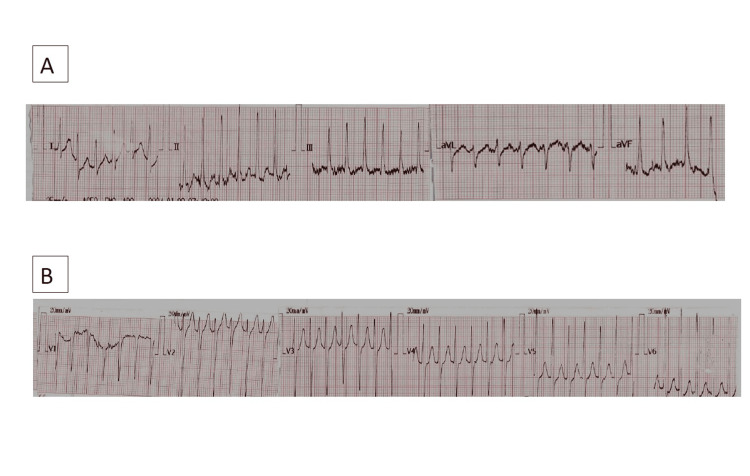
(A) ECG showing supraventricular tachycardia (SVT) with narrow QRS complexes and obscured P waves in limb leads. (B) Corresponding precordial (chest) leads showing the same tachyarrhythmia pattern.

**Figure 3:**
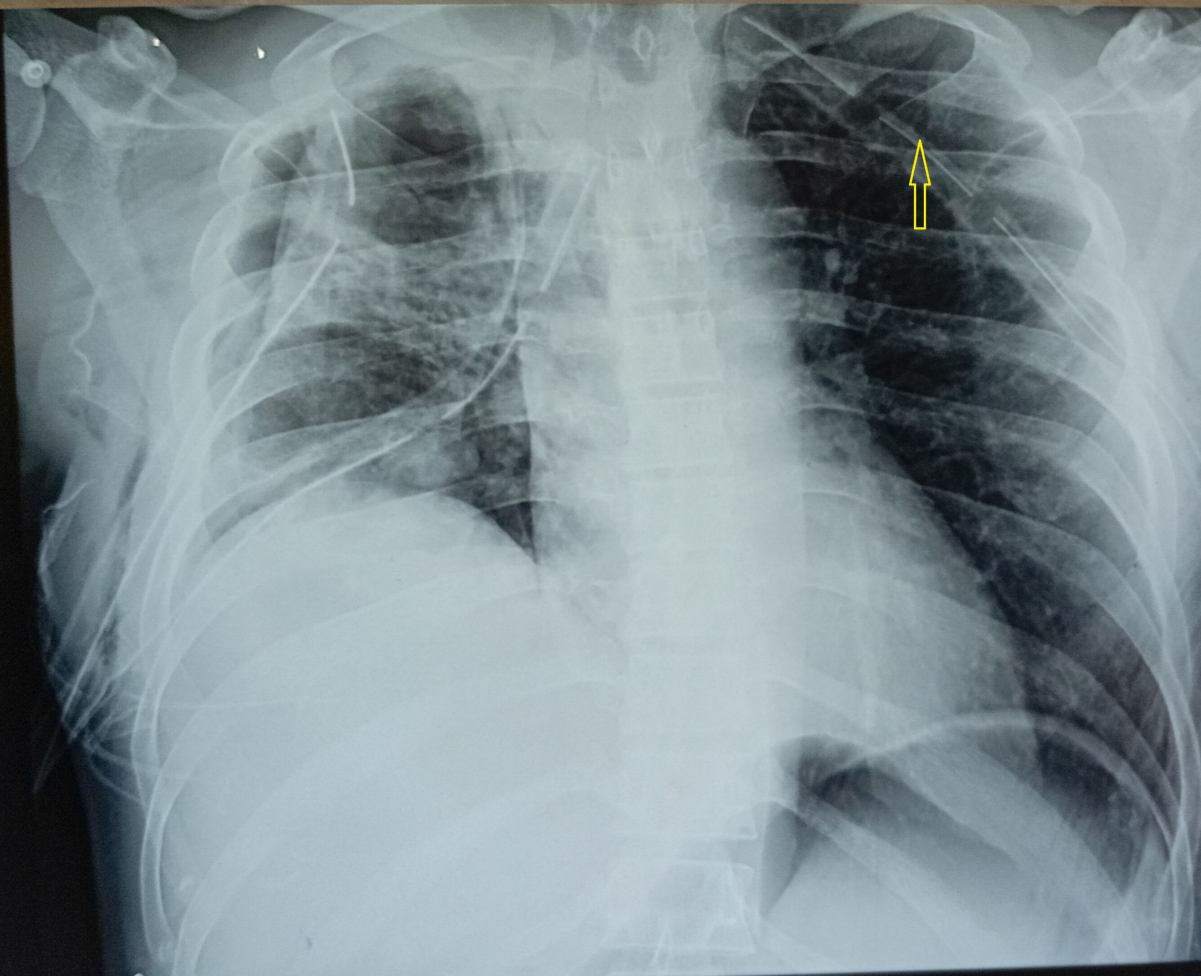
Chest X-ray showing multiple intercostal tubes; left sided tube adjacent to the mediastinum (yellow arrow).

The patient’s symptoms improved following drainage. Approximately 36 hours after placement of the left-sided tube, on the fourth day of admission, the patient developed a sudden onset of palpitations and dyspnea. Examination revealed tachycardia with a heart rate of 193 beats per minute, blood pressure of 94/72 mmHg, a respiratory rate of 40 breaths per minute, and oxygen saturation of 92% on room air. Electrocardiogram (ECG) revealed supraventricular tachycardia (SVT) (Figure 3). He was initially treated with carotid sinus massages, which were ineffective. He then received two doses of intravenous adenosine 6mg, followed by a single 12 mg dose, after which he reverted to sinus rhythm (Figure 2). Oral verapamil 40 mg, eight eight-hourly doses, was commenced as a precautionary antiarrhythmic to prevent recurrence.

Investigations were performed to identify the cause of SVT. Serum electrolytes, cardiac troponin 1, thyroid function tests, and sepsis screening (sputum, blood, and urine cultures) were all within normal limits. A 2D echocardiogram showed no structural cardiac abnormalities. A repeated chest X-ray demonstrated that the left-sided intercostal tube was abutting the mediastinum (Figure 3), suggesting mechanical irritation as a potential trigger for the arrhythmia. Chest computed tomography (CT) was considered for more definitive evaluation; however, it was not performed due to clinical improvement and resource limitations. The left intercostal tube was removed approximately 24 hours after SVT onset, after which the patient had no further episodes of arrhythmia. Verapamil was discontinued five days after tube removal. He remained in sinus rhythm throughout the remainder of hospitalization and during outpatient follow-up.

Subsequent investigations confirmed pulmonary tuberculosis based on a positive sputum GeneXpert test. Antituberculous therapy was initiated according to national guidelines, and the patient is currently under regular follow-up at the chest clinic.

## Discussion

Supraventricular tachycardia (SVT) is a common arrhythmia encountered in clinical practice. Causes range from idiopathic to secondary triggers such as structural heart disease, autonomic dysregulation, and post-surgical irritation. In this case, SVT occurred in a previously healthy adult following intercostal (IC) tube insertion, raising suspicion of a mechanical trigger due to mediastinal contact.

The mediastinum contains critical cardiac autonomic plexuses, vascular structures, and pericardium - all of which may be sensitive to external irritation. Chest radiography revealed the IC tube positioned medially, close to the cardiac silhouette. Although the precise anatomical relationship between the IC tube and left atrium could not be delineated on the chest X-ray alone, the proximity raises the possibility of mechanical irritation as a plausible contributory factor. This aligns with case reports of arrhythmia occurring secondary to catheter or chest tube-related cardiac contact during surgery or trauma [3,4]. 

It is important to acknowledge, however, that SVT, especially atrioventricular nodal reentrant tachycardia (AVNRT), can occur in patients without structural heart disease or external triggers. The onset of SVT in this case may have been coincidental or related to other unrecognized factors such as stress or hypoxia. Nevertheless, the timing of the onset of the lung re-expansion phase in which thoracic anatomy shifts dynamically [5], and the absence of other precipitating abnormalities (electrolyte imbalance, infections, or preexisting heart disease), supports a possible mechanical etiology.

The patient was initially managed with oral verapamil (40mg every eight hours), which effectively controlled the arrhythmia. Verapamil was discontinued after the resolution of SVT and removal of the IC tube, and the patient remained free of recurrence during follow-up. This suggests that pharmacological therapy was only required transiently, and that the arrhythmia may have been self-limiting once the mechanical factor was addressed.

From a clinical perspective, this case highlights the importance of careful IC tube placement, particularly avoiding medial angulation that may impinge upon mediastinal or cardiac structures. Post-procedural imaging should be carefully reviewed to assess high-risk positioning. In selected cases where arrhythmias developed after IC tube insertion, consideration should be given to mechanical causes as a differential diagnosis.

## Conclusions

This case illustrates that supraventricular tachycardia (SVT), although not exclusive to chest tube insertion, may arise due to mechanical irritation when a tube is positioned close to the mediastinum and adjacent cardiac structures. While a stressful clinical context and pneumothorax itself can precipitate arrhythmias following intercostal tube placement, clinicians should consider the potential role of physical irritation.

To minimize the risk, it is advisable to avoid mediastinal positioning of chest tubes and routinely confirm placement using imaging to ensure the drain does not abut the heart or greater vessels. Continuous ECG monitoring in complex thoracic cases may facilitate early detection of arrhythmias. Although SVT is a common arrhythmia, its occurrence in the specific context of mechanical irritation by a chest tube remains infrequently reported and poorly understood. Additional case reports and prospective data are necessary to further evaluate the true incidence, mechanisms, and risk factors of this phenomenon. 
